# CD8^+^ CD226^high^ T cells in liver metastases dictate the prognosis of colorectal cancer patients treated with chemotherapy and radical surgery

**DOI:** 10.1038/s41423-023-00978-2

**Published:** 2023-01-30

**Authors:** Julien Viot, Syrine Abdeljaoued, Angélique Vienot, Evan Seffar, Laurie Spehner, Adeline Bouard, Kamal Asgarov, Jean-René Pallandre, Elodie Renaude, Elodie Klajer, Chloé Molimard, Franck Monnien, Frederic Bibeau, Celia Turco, Bruno Heyd, Paul Peixoto, Eric Hervouet, Romain Loyon, Alexandre Doussot, Christophe Borg, Marie Kroemer

**Affiliations:** 1 Department of Medical Oncology, Biotechnology and Immuno-Oncology Platform, University Hospital of Besançon, Besançon, France; 2 INSERM, EFS BFC, UMR1098, RIGHT, University of Franche-Comté, Interactions Greffon-Hôte-Tumeur/Ingénierie Cellulaire et Génique, Besançon, France; 3 Department of Pathology, University Hospital of Besançon, Besançon, France; 4 Department of Surgery, University Hospital of Besançon, Besançon, France; 5 EPIGENEXP platform, University of Bourgogne Franche-Comté, Besançon, France; 6 Department of Pharmacy, University Hospital of Besançon, Besançon, France

**Keywords:** CD226 antigen, Colorectal Neoplasms, Liver metastasis, Lymphocytes, Tumor-Infiltrating, Gastrointestinal cancer, Prognostic markers, Cancer microenvironment, Immune evasion, Cytotoxic T cells

## Abstract

CD226 has been reported to participate in the rescue of CD8^+^ T cell dysfunction. In this study, we aimed to assess the prognostic value of CD226 in tumor-infiltrating lymphocytes (TILs) derived from colorectal cancer (CRC) liver metastases treated with chemotherapy and radical surgery. TILs from 43 metastases were isolated and analyzed ex vivo using flow cytometry. CD155 and CD3 levels in the tumor microenvironment were assessed by immunohistochemistry. Exploration and validation of biological processes highlighted in this study were performed by bioinformatics analysis of bulk RNA-seq results for 28 CRC liver metastases pretreated with chemotherapy as well as public gene expression datasets. CD226 expression contributes to the definition of the immune context in CRC liver metastases and primary tumors. CD226 on CD8^+^ T cells was not specifically coexpressed with other immune checkpoints, such as PD1, TIGIT, and TIM3, in liver metastases. Multivariate Cox regression analysis revealed CD226 expression on CD8^+^ T cells to be an independent prognostic factor (*p* = 0.003), along with CD3 density at invasion margins (*p* = 0.003) and TIGIT expression on CD4^+^ T cells (*p* = 0.019). CD155 was not associated with the prognostic value of CD226. Gene expression analysis in a validation dataset confirmed the prognostic value of CD226 in CRC liver metastases but not in primary tumors. Downregulation of CD226 on CD8^+^ TILs in the liver microenvironment was restored by IL15 treatment. Overall, CD226 expression on liver metastasis-infiltrating CD8^+^ T cells selectively contributes to immune surveillance of CRC liver metastases and has prognostic value for patients undergoing radical surgery.

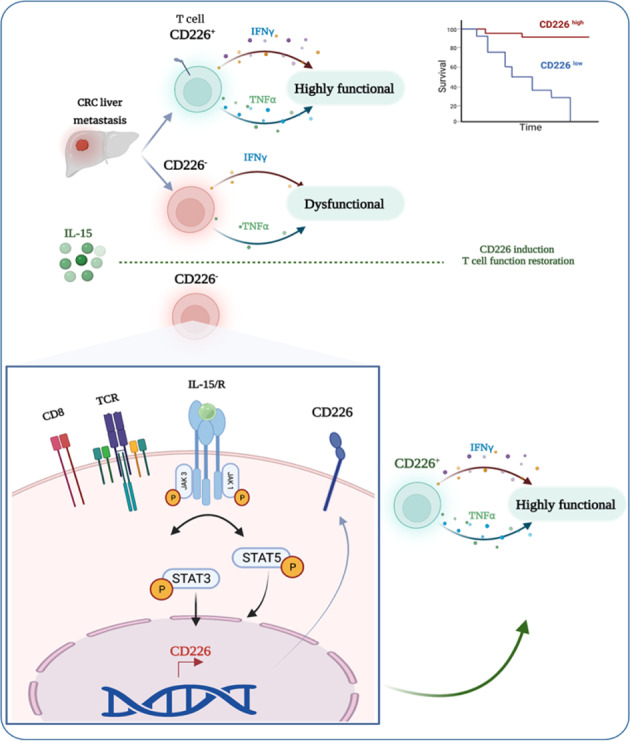

## Background

Despite the progress achieved over the past decade, the combination of more effective therapeutics and increased rate of liver-confined metastasis resection, metastatic colorectal cancer (mCRC) remains among the top three leading causes of cancer-related death [1]. Indeed, over half of colorectal cancer patients develop recurrent liver metastases within two years [2]. Prognostic biomarkers in primary colorectal cancer (CRC) have been widely explored; however, the determinants of metastasis remain unclear.

The degree of lymphocytic infiltration, as well as its phenotype, dictates clinical outcomes in primary and liver metastases [3, 4]. Although tumor immune infiltration in primary CRC can affect prognosis and response to therapy [5], the liver is a unique immunological organ with a strong intrinsic immune suppression environment that might potentiate mechanisms related to cancer immune evasion [6]. In primary tumors, CD3 infiltration, MHC class I expression, and the IFNy signature are good determinants of potent antitumor activity [7]. Nevertheless, only a few studies on metastasis have been conducted [8], and the immune context of primary and mCRC might differ [9]. For a better understanding of the immune landscape in the cancer microenvironment, further elucidation of cytotoxic lymphocyte subsets directly invading CRC metastases and their cognate modes of activation or exhaustion is strongly warranted.

CD226 (DNAM-1, DNAM1, PTA1, and TLiSA1) and TIGIT are mainly expressed on NK and T cells and contain intracellular activation and inhibitory domains, respectively [10]. These immune receptors bind to their ligands CD155 (PVR, necl-5) and CD112 (nectin-2) competitively [11, 12]. CD226 is a marker of highly functional T cells and contributes to cancer immune surveillance by NK cells [13]. TIGIT is upregulated and coexpressed with PD1 in exhausted T cells, and antibody-mediated blockade of TIGIT restores the antitumor immunity of CD8^+^ T cells [14], and the effects of TIGIT blockade are likely related to CD226 expression [15].

CD8^+^ T cells exhibiting high CD226 expression show upregulated production of cytokines (such as IL13, IL10, IL17, and IFNy) and cytotoxicity [16, 17]. The role of CD226 in CD8^+^ T cell function has recently been described in the context of cancer [18, 19]. CD226 is not required for antigen presentation, and there are no immune defects in CD226-deficient mice [15]. Nonetheless, CD226 has been identified as a costimulatory molecule that participates in the rescue of CD8^+^ T cell dysfunction and maintains the memory phenotype [18, 20]. Furthermore, CD226 has been reported to correlate with a Th1 phenotype in CD4^+^ T cells, regulating their expansion, as well as their effector functions, such as IFNy production [21, 22]. Downregulation of CD226, owing to both an EOMES-dependent transcriptional mechanism and CD155-mediated posttranslational mechanism, limits the efficacy of immune checkpoint inhibitors [18, 20, 23].

CD226 expression by NK and T cells has been investigated in several cancer types, such as breast cancer, melanoma, pancreatic adenocarcinoma, and myeloma [11, 13, 18]. In pancreatic cancer, CD8^+^CD226^high^ T cells are considered to be predictive biomarkers of immune efficacy [15].

To the best of our knowledge, the precise role and clinical impact of CD226 expression on tumor-infiltrating lymphocytes (TILs) in liver metastases has never been investigated. We hypothesized that CD226 is a marker of nonexhausted T cells that maintains potent cytotoxic activity and increases survival rates when expressed by TILs in liver metastases. To test this hypothesis, TILs from 43 liver metastases of 39 patients were isolated and thoroughly analyzed ex vivo using multiparametric flow cytometry and immunostaining. Findings related to CD226 and CD155 were validated in public gene expression datasets of primary CRC and liver metastases and by using a local RNA-seq dataset of 27 liver metastases from CRC treated with chemotherapy.

## Methods

### Patients and healthy donor-derived materials

All tumors used as samples were excised by surgeons from the Department of Digestive Surgery at the University Hospital of Besançon between May 2016 and July 2019. All patients were enrolled in the Epitopes-CRC02 (NCT02817178) cohort after obtaining informed consent in accordance with French regulations and approval by the local and national ethics committees. Peripheral blood samples were collected from 16 patients in the cohort prior to surgery. Paraffin-embedded tissue specimens from each patient were retrieved from pathology archives and processed/archived in Biobank BB-0033-00024, Tumorothèque Régionale de Franche-Comté, throughout the project (#F1820). Liver metastases and paired primary tumors (when available) from 39 CRC patients treated with chemotherapy were analyzed using RNA next-generation sequencing.

### Tumor processing for TIL extraction

TIL extraction from CRC liver metastases was based on a method previously established by Dudley et al. [24]. Briefly, tumors were cut, digested using a mechanical/enzymatic dissociation system (Miltenyi Biotec), and filtered. The resulting cell suspension was subjected to immunofluorescence staining for flow cytometry. Further details are provided in the Supplementary Methods.

### T cell culture and functional assays

Tumor-infiltrating lymphocytes were stimulated for 5 h with plate-bound anti-CD3 (Miltenyi Biotec MACS GMP, clone OKT3) and soluble anti-CD28 (Miltenyi Biotec, clone 15E8) antibodies in the presence of GolgiPlug (BD Biosciences) in RPMI 1640 medium (Thermo Fisher Scientific) supplemented with 10% fetal bovine serum. After stimulation, intracellular cytokine staining was performed to assess IFNy (BD Biosciences, clone B27) and TNFα (BD Biosciences, clone Mab11). For CD226 downregulation and induction assays, cells were stimulated for 6 days with TGF-β (50 ng/ml, Peprotech), IL15 (50 ng/ml, Miltenyi Biotec) or IL2 (20 UI/ml, Roche). For STAT inhibitor experiments, cells were incubated for 48 h with IL15 in the presence of STAT3i (CAS 501919-59-1, Sigma‒Aldrich) or STAT5i (CAS 2062-78-4, Sigma‒Aldrich).

### Flow cytometry

Before immunostaining, peripheral blood mononuclear cells (PBMCs) and TILs were counted and suspended in FACS buffer (1X PBS, 50 µM EDTA, 0.2% BSA) before labeling with appropriate antibodies for 30 min in the dark at 4 °C. The cells were then washed with 1X PBS. To determine viability, the cells were stained with eFluor 780 viability dye (eBioscience) according to the manufacturer’s instructions.

The following antibodies were used: Pacific Blue anti-CD3 (UCHT1), BV510 CD8 (SK1), FITC/PerCp-Cy5.5 CD226 (11A8), PercP/Cy5 anti-CD4 (clone OKT4), Pe/Cy7 anti-PD1 (clone EH12.2H7), A700 CD69 (FN50), BV711 CD103 (Ber-ACT8), and PE anti-TIM3 (F38-2E2) (clone MBSA43). Samples were acquired using a FACS Canto II or FACSLyric (BD Biosciences) and analyzed with FACSDiva software (BD Biosciences). The gating strategy was set on isotype controls applied in a standard format across all samples.

### Immunohistochemistry

Archival formalin-fixed and paraffin-embedded specimens were sectioned at 4/5 μm. The first section was histopathologically assessed by HES staining to confirm the presence of tumor cells. Immunostaining for CD3 with a polyclonal rabbit anti-human CD3 antibody (A045201, Agilent) [1:200] was performed using a Ventana Benchmark (Roche). A polyclonal antibody against CD155 (anti-poliovirus/PVR; Abcam) was utilized to detect CD155. The relevant protocols are described in the Supplementary Material. CD3 slides were digitized with a Nanozoomer HT2.0 (Hammamatsu) at 20× magnification to generate a whole-slide imaging (WSI) file in ndpi format and imported into QuPath software [25] to evaluate densities (cells/mm²) of CD3 in the CT and IM. CD155 expression was defined as membranous staining of tumor and bill duct cells. Immunohistochemical staining was performed by a pathologist who was blinded to patient identity or clinical status.

### Chromatin immunoprecipitation (ChIP)

Chromatin was prepared with truChIP™ Chromatin Shearing Kit (Covaris, Brighton, UK) according to the manufacturer’s instructions. Each sample was sonicated for 12 min using a Covaris sonicator E220 Evolution. ChIP was performed using the IP-Star Compact Automated System (Diagenode, Liege, Belgium). Briefly, 6 µg of isolated chromatin was immunoprecipitated with either 600 ng ChIP grade antibody anti-SMAD2/3 (5678, Cell Signaling) or 600 ng of IgG (C15410206, Diagenode, Liege, Belgium) in dilution buffer (0.01% SDS; 1.1% Triton X 100; 2 mM EDTA; 16.7 mM Tris-Cl pH 8.0; 167 mM NaCl; 1× protease inhibitor cocktail, Sigma Aldrich, St Louis, MI, USA). The DNA/protein complexes were washed two times in IP Wash buffer 1 (20 mM Tris-HCl pH 8.0, 150 mM NaCl, 1%; Triton X100), one time in IP Wash buffer 2 (20 mM Tris-HCl pH 8.0, 450 mM NaCl, 1%; Triton X100) and one time in IP Wash buffer 3 (20 mM Tris-HCl pH 8.0; 500 mM LiCl; 1%; NP40, 0.5% deoxycholic acid). After reversal of crosslinking, the immunoprecipitated DNA was purified using a Chromatin IP DNA Purification kit (58002, Active Motif) and analyzed by RT‒qPCR with SYBR-Green Takara (Ozyme, Paris, France); a real-time PCR system was applied in step one. The PCR conditions were 10 min at 95 °C followed by 45 cycles of 10 s at 95 °C, 30 s at 60 °C, and 30 s at 72 °C. The following primers were used: promoter CD226 [F] 5’-GAAGAGGTGAAAGGAAGGCCA-3’ and [R] 3’-GCATAAGATGAGGCAGAGGC-5′.

### RNA-seq preparation

Frozen samples from liver metastases of CRC retrieved surgically from 2009 to 2019 were used for mRNA extraction using Qiagen Kit Qiagen AllPrep DNA/RNA Mini Kit (ref 80204). The concentration of each library was measured by real-time PCR, and samples with an RIN score of < 7 were discarded. The constructed libraries were sequenced (200 cycles) in paired ends (2 × 100 bp) using Novaseq 6000.

### Bioinformatic analysis

Normalized RNA-seq fragments per kilobase million (FPKM) (GRCh38) of 478 colon adenocarcinoma patients from The Cancer Genome Atlas (TCGA) were retrieved using the R package TCGAbiolinks [26].

Affymetrix DNA expression sets from the GPL570 platform (GSE14333, GSE17536, GSE33113, GSE37892, and GSE39582) focusing on primary CRC were retrieved from public repositories. Data were merged and linked to patient survival. Mean expression of CD155 was determined for each sample using the four probes with the best variance and similar mean expression. Analyses were discretized into two groups (high and low) based on median expression of CD226 and CD155. Relapse-free survival (RFS) analysis was performed using 1046 patients and overall survival (OS) for 737 patients using the Survminer R package according to the high and low expression groups.

Bulk RNA-seq data from 54 samples (normal colon, primary CRC, and CRC liver metastasis) generated for 18 CRC patients were retrieved from Gene Expression Omnibus (GEO) GSE50760 [27] using normalized FPKM.

Local bulk RNA-seq reads were aligned using STAR v2.7.9a to GENCODE Human Release 39 (GRCh38.p13), and the read count matrix was produced with FeatureCounts v2.0.1, both by using the default parameters.

Immune deconvolution analyses of the datasets were performed using the immunedeconv R package [28] and MCPCounter [29]. Clustering of the CD226^high^ and CD226^low^ expression populations was performed by cutting off the median of full CD226 gene expression for the dataset. A gene signature score was calculated using the geometric mean of log2(1+ gene expression matrix (FPKM-normalized)) transformation.

The single-cell RNA-seq dataset was collected from Gene Expression Omnibus accession number GSE178318 [30]. Quality control and downstream analysis were performed in Seurat v4.0.6 [31] to remove cells with a low number of uniquely detected genes (< 500) and discard cells in which the proportion of the UMI count attributable to mitochondrial genes was greater than 10%, resulting in an overall of 107820 remaining cells and 23289 genes. Batch integration of the samples was performed with Harmony [32] using 2000 highly variable genes. The annotation of each cell cluster was realized using a list of gene markers delivered in the source data article, and our analysis focused on T cells from liver metastases and primary tumors expressing the following gene markers: CD3G, CD3D, CD8A, and CD226. Differentially expressed gene analysis was performed using the Seurat integrated function ‘Find Markers’ to compare cells expressing the CD226 marker and those not expressing it among CD8 cells. We selected the most significant genes with an adjusted p value below or equal to 0.05 and a Log2FoldChange below -0.25 and above 0.25.

TCR analysis was performed on the bulk RNA-seq data using MiXCR v3.0.3, with the single command parameter “shotgun” for the entire pipeline.

Computations were performed at the supercomputer facilities of *Mésocenter de calcul de Franche-Comté*.

Raw data from the bulk RNA-seq analysis were deposited in NCBI Gene Expression Omnibus (GEO) under accession number GSE207194.

### Statistical analysis

Median (interquartile range) and frequency (percentage) values are used to describe continuous and categorical variables, respectively. Medians and proportions were compared using Student’s t test and the chi-square test (or Fisher’s exact test, if appropriate), respectively.

OS was calculated from the date of liver metastasis surgery to the date of death from any cause. RFS was calculated from the date of surgery for liver metastasis to the date of relapse or death from any cause or the date of the last follow-up, at which point the data were censored. Survival data were censored at the last follow-up visit. OS and RFS were estimated using the Kaplan‒Meier method and are described using the median or rate at specific time points with 95% confidence intervals (95%CI).

The Cox proportional hazard model was employed to estimate the hazard ratio (HR) and 95% confidence interval (CI) for factors associated with RFS. The association of parameters with RFS was first assessed using univariate Cox analyses, and then parameters with *p* < 0.05 were entered into a final multivariate Cox regression model.

All analyses were performed using R software (version 3.6.1; R Development Core Team; http://www.r-project.org) and GraphPad Prism v9.3.1. Statistical significance was set at *P* < 0.05, and all tests were two-sided.

## Results

### CD226 is associated with a potent immune contexture in CRC leading to better prognosis for liver metastatic diseases

Although the role of CD226 in TCR signaling and CD8^+^ T cell functions has been recently established [18], the role of this receptor in the CRC immune context remains elusive. We first sought to gain insight into the contribution of CD226 to immune-related gene expression in both primary tumors and liver metastases from CRC.

**Fig. 1:**
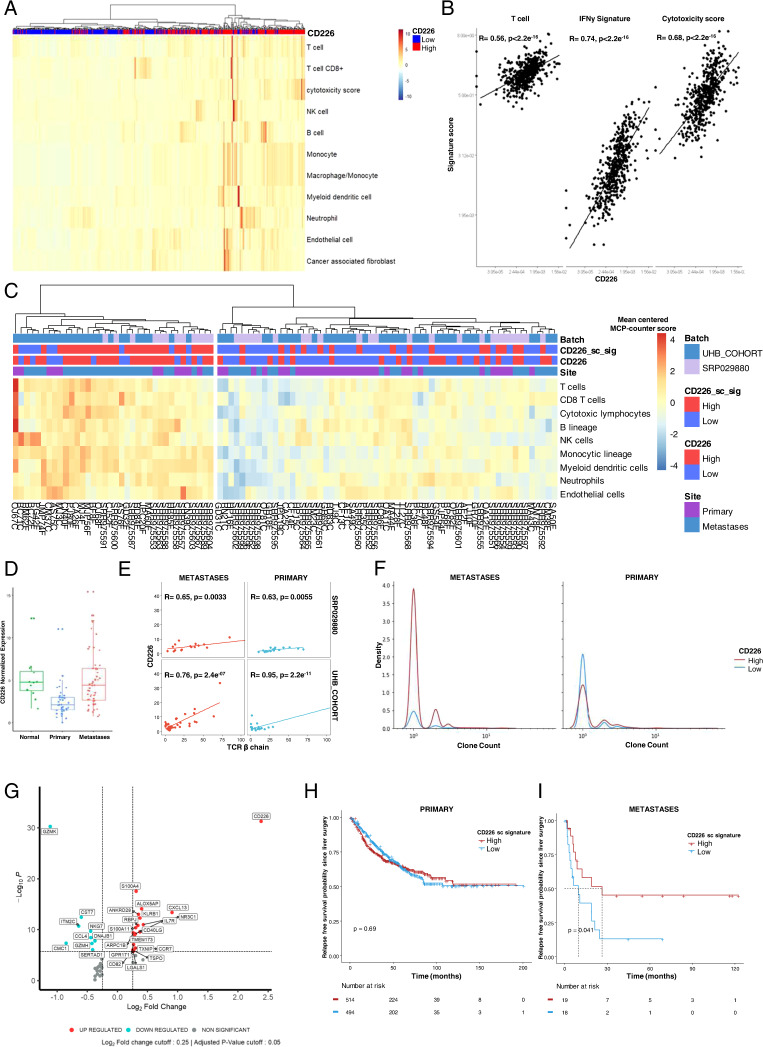
CD226 is associated with a potent immune contexture in CRC, leading to a better prognosis in liver metastases. **A** Immune deconvolution using MCPcounter for RNA-seq data for primary CRC from TCGA. Annotation of the CD226 expression level discretized from median expression in the whole cohort. **B** Correlation of CD226 and signature scores for T cells and cytotoxicity using MCPcounter for RNA-seq data for primary CRC from TCGA and the interferon gamma secretion signature: *IFNG, CD274, LAG3, CXCL9*. **C** Immune deconvolution using MCPcounter for RNA-seq data for paired primary and liver metastasis from 39 patients from University Hospital of Besancon and 18 patients from public database *GSE50760/SRP029880*. Annotation according to sample site, CD226 expression level discretized from median expression in the whole cohort and CD226^+^ T cell signature from single-cell RNA-seq described in **G**. **D** CD226 normalized expression level (TMM) from previous cohorts for normal adjacent colon tissue, primary CRC and CRC liver metastasis. **E** Correlation between CD226 expression level and number of distinct beta chains of the TCR calculated for each sample with MiXCR. The number of beta chains of the TCR is a reflection of TCR diversity. **F** Frequency of clone counts from the beta chain of the TCR according to the level of CD226 discretized from median expression in the whole cohort and sample site. **G** Volcano plot of differential expression of T cells in liver metastases from CRC of public single-cell RNA-seq GSE178318 according to CD226^+^ versus CD226^-^. Log2-fold-change cutoff of 1 and adjusted p cutoff of 0.05. **H** Estimated RFS since surgery according to the CD226 signature created from previous differential analysis in a *“home curated”* database of primary CRC. The score was calculated from the geometric mean of the mRNA expression level from the gene list. For survival analysis, patients were divided into two groups based on the median value of all CD226 signature scores. **I** Kaplan‒Meier curve for RFS probability since surgery for liver metastasis in the University Hospital of Besancon cohort according to the gene signature for CD226-associated T cells with the same method as **H**. For survival analysis, patients were divided into two groups based on the median value of all CD226 signature scores. The Pearson correlation coefficient is shown as R, with p values from the correlation test. On survival graphs, *p* based on the log-rank test. CRC Colorectal cancer, RFS Relapse-free survival

We first examined the association of CD226 with the immune environment using public RNA-seq datasets. Bulk gene expression analysis revealed low expression of CD226 in both the normal colon and liver (transcript per million (TPM) < 1). In contrast, CD155 was highly expressed (TPM > 14) in these organs. CD226 expression levels were similar in the colon and in the liver, whereas CD155 expression was higher in normal liver than in normal colon (Supplementary Fig. S1A). CD155 RNA was expressed at twice the rate in CRC tissues as in normal tissues. In contrast, CD226 displayed similar expression patterns in primary CRC and normal tissues (Supplementary Fig. S1B). Next, we explored the correlation between ligands and receptors of the CD226/TIGIT axis using a dataset from TCGA. Receptors (CD96, CD226, TIGIT, and PVRIG) correlated positively with each other. Ligands correlated negatively with receptors, with the best association between CD226 and CD155 (Pearson’s correlation coefficient = −0.3). Some nonsignificant associations between CD112 and TIGIT were also observed (Supplementary Fig. S1C). These findings were also validated in an independent cohort. The RNA-seq dataset from TCGA was then used to validate the correlation between CD226 expression and immune cell infiltration transcriptomic profiles in primary CRC. We observed a strong association between CD226^high^ expression and an inflamed immune context (Fig. 1A). All immune cell signatures identified with MCPcounter were found to be overexpressed in the CD226^high^-enriched cluster. Analysis of primary CRC immune activation using T cells, interferon-gamma secretion, and cytotoxicity score signatures revealed a correlation with CD226 gene expression (Pearson’s correlation coefficients were 0.56, 0.74, and 0.68, respectively; *p* < 0.05) (Fig. 1B). Therefore, CD226 is associated with a potent immune response in primary CRC.

To investigate CD226 expression in liver metastases, the paired primary cancers and liver metastases of 18 patients from GSE50760 and 39 patients from the University Hospital of Besancon (UHB cohort) were analyzed. CD226 expression was enhanced in CRC metastases compared to primary tumors and correlated with an inflamed immune environment (Fig. 1C). CD226 expression from liver metastases displayed significantly higher RNA expression compared to primary CRC (*p* = 0.001). However, CD226 gene expression levels were widely distributed in liver metastases. Altogether, there was no difference between median CD226 expression in liver metastases and normal tissue, suggesting downregulation of CD226 in primary CRC in our cohort (Fig. 1D). TCR sequencing was performed on the samples using MiXCR. There was a strong correlation between CD226 expression and the number of TCR β chains identified in the samples, suggesting that CD226 was associated with a high diversity in the TCR repertoire in both cohorts (Fig. 1E). Next, we investigated the frequency of TCR clones based on CD226 expression. Interestingly, although no difference was observed in primary CRC, high CD226 expression was associated with increased TCR clone counts in liver metastases (Fig. 1F), suggesting that CD226 is associated with an immune context in which T cell polyclonal amplification is possible.

We then took advantage of a single-cell RNA-seq dataset to investigate gene expression levels according to CD226 expression in TILs isolated from CRC. We first explored differential gene expression by analyzing T cells from primary and liver metastases. Ten genes were upregulated (IL7R, CD40LG, and CXCL13) and five downregulated (GZMK) (Supplementary Fig. S1D). The same differential analyses were performed for T cell-infiltrating liver metastases from patients with CRC. Five genes showed downregulated expression (DNAJB1, SERTAD1, GZMH, CCL4, and ITM2C) and twelve upregulated (CXCL13, NR3C1, RBPJ, S100A4, S100A11, CCR7, TXNIP, GPR171, ARPC1B, TSPO, LGALS1, CD82) in TILs expressing CD226 derived from liver metastases (Fig. 1G). All genes correlating with CD226 in CRC TILs were used as signatures and tested for prognostic impact using bulk RNA-seq from primary CRC. Additionally, a CD226-related gene expression signature was applied to a bulk RNA-seq dataset derived from the liver metastases of CRC patients (UHB cohort).

A dataset of 737 patients with primary CRC from five Affymetrix DNA expression sets of GPL570 was manually curated to obtain survival data. There was no difference in RFS probability in localized CRC according to CD226-related gene expression signature level (Cox regression: HR = 0.957, 95% CI = 0.848–1.066, *p* = 0.691) (Fig. 1H). In addition, we observed no differences in OS (*p* = 0.47) (Supplementary Fig. S1E). In contrast, the CD226 signature discriminated RFS in the exploratory UHB cohort dataset, which included CRC liver metastases. Low expression of the CD226-related signature correlated with decreased RFS (9.66 months [95% CI = 4.5–24.6 months] vs. 26.38 months [95% CI = 8.71 to NA months] in the CD226^high^ group (*p* = 0.041, Fig. 1I). A similar impact of the CD226-related signature was observed for OS (median OS of 31.6 months [95% CI = 21.7 to NA months] versus 68.6 months [95% CI = 41.3 to NA months], *p* = 0.069; Supplementary Fig. S1F).

Taken together, these preliminary results suggest that the immune context related to CD226 expression differs between primary CRC and CRC liver metastases. Therefore, a deeper characterization of TILs according to CD226 status was conducted for liver metastasis.

### CD226 expression is downregulated on CD8^+^ TILs derived from CRC liver metastases

**Fig. 2:**
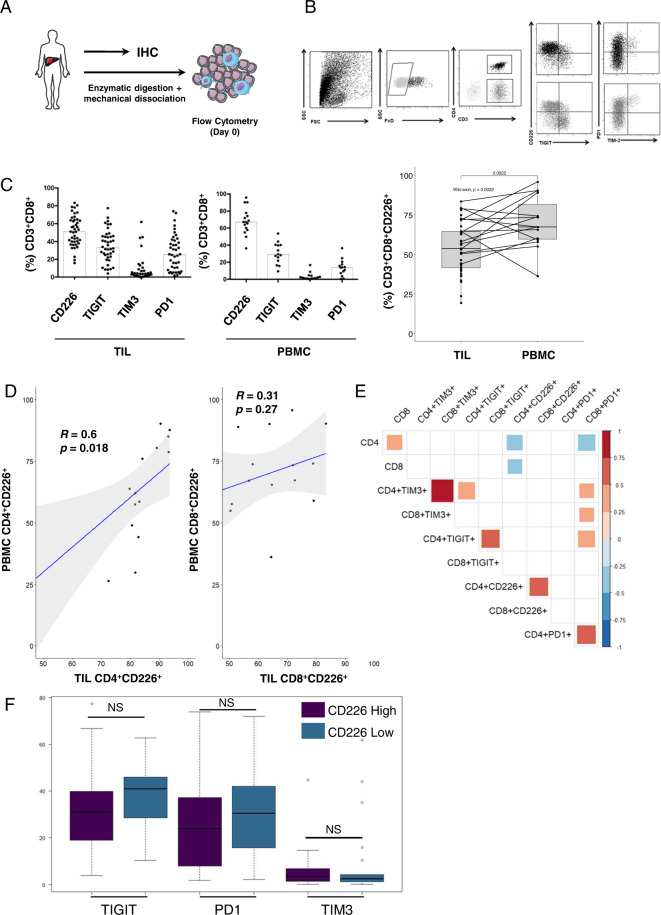
Description of CD226 expression on TILs and matched peripheral T lymphocytes. **A** Model description: pieces of liver metastasis from CRC, as removed by surgery, were used for immunohistochemistry or dissociated mechanically and enzymatically for flow cytometry analysis of TILs. Forty-three TILs from 39 patients were analyzed. PBMCs for 16 of the 39 patients were available for comparison with TILs. **B** Flow cytometry gating strategy: gating on alive and singlets, then for CD226, TIGIT, TIM3 and PD1 expression on CD8^+^ and CD4^+^ T cells. **C** Checkpoint expression on CD8^+^ T cells in TILs and PBMCs. The percentage of CD8^+^ T cells positive for receptors is represented on the left. On the right, paired plot representing the percentage of CD3^+^CD8^+^CD226^+^ cells between TILs and PBMCs. **D** Correlation of the respective proportions of CD4^+^CD226^+^ and CD8^+^CD226^+^ cells between PBMCs and TILs. The Pearson correlation score is represented as *R*, and the p value is represented as *p*. **E** Correlation matrix between checkpoints CD226, PD1, TIGIT and TIM3 on CD4^+^ and CD8^+^ TILs. Significant correlations are marked in a color gradient according to their value. Nonsignificant associations are shown in white. *p* < 0.05 was considered significant. **F** PD1, TIGIT and TIM3 expression on CD8^+^ cells between two populations: CD226^high^ versus CD226^low^ on CD8^+^ T cells. Discretization in the high or low group is according to CD226 expression based on median expression according to cytometry for the full dataset. CRC Colorectal cancer, NS Not significant. PBMCs Peripheral blood mononuclear cells, TILs Tumor-infiltrating lymphocytes

CD226 expression defines phenotypically and functionally distinct CD8^+^ T cell subsets among both TILs and matched peripheral T cells [18]. As the expression pattern of CD226 has never been investigated in CRC-derived metastases, we sought to better characterize CD226-expressing lymphocytes in 43 liver metastases from 39 CRC patients. Multiparametric flow cytometry was performed ex vivo using fresh and uncultured cells immediately after surgery. PBMCs from 16 patients were retrieved on the day of surgery (Fig. 2A). After excluding dead cells and doublets, the flow cytometry gating strategy involved immune checkpoint (CD226, TIGIT, PD1, TIM3) expression on CD4^+^ and CD8^+^ TILs (Fig. 2B).

We observed strong expression of CD226 on CD4^+^ TILs across samples (mean positivity 72.7% 1st Qu 65.5%; 3rd Qu 85.2%) and moderate CD226 levels on CD8^+^ TILs (mean positivity 51.1% 1st Qu 39.8%; 3rd Qu 64.1%) (Supplementary Fig. 2, Fig. 2C). The immune checkpoints PD1 and TIM3, but not CD226 or TIGIT, were overexpressed on CD4^+^ TILs compared with peripheral T cells [mean expression: 22.2% vs. 11.9%, *p* = 0.008; 6.2% vs. 1.5%, *p* = 0.004; 72.7% vs. 62.2%, *p* = 0.069; 19.7% vs. 14.8%, *p* = 0.057, respectively]. Although TIGIT (mean expression: TILs 35% vs. PBMCs 30.4%, *p* = 0.297) and TIM3 (TILs 7.9% vs. PBMCs 3.5% *p* = 0.071) displayed a similar distribution between TILs and peripheral T cells, increased expression of PD1 (TILs 27.2% vs. PBMCs 13.5% *p* = 0.001) was observed on CD8^+^ TILs compared to peripheral CD8^+^ T cells (Fig. 2C). Moreover, CD8^+^ TILs exhibited a significant decrease in CD226 expression compared to peripheral T cells [mean expression: 51.7% vs. 68.1%, *p* = 0.0022]. Notably, a correlation was observed between CD226 expression on CD4^+^ T cells assessed on matching TILs and PBMCs (Pearson’s correlation coefficient of 0.6 and *p* = 0.018) but not on CD8^+^ T cells (Pearson’s correlation coefficient of 0.31 and *p* = 0.27) (Fig. 2D). These observations led us to speculate that loss of CD226 expression on CD8^+^ T cells might be driven by the tumor microenvironment. Notably, increased expression of PD1 on TILs, reminiscent of antigen encounters, was observed for both CD226^+^ and CD226^−^ CD8^+^ T cells.

Using a correlation matrix including all immune checkpoints investigated in previous experiments, we observed that CD226 expression on CD4^+^ and CD8^+^ TILs did not correlate with PD1, TIM3, or TIGIT expression by these lymphocytes. Conversely, TIGIT correlated positively with TIM3 and PD1 expression. Immune checkpoint expression also correlated with CD4^+^ and CD8^+^ TILs, with correlation coefficients of 0.61, 0.46, 0.7, and 0.54 for CD226, PD1, TIM3, and TIGIT, respectively (Fig. 2E).

Next, we studied TIM3, TIGIT, and PD1 expression on TILs based on the median expression level of CD226. There was no significant difference in PD1, TIGIT, and TIM3 expression between CD226^high^ and CD226^low^ CD8^+^ T cells (Fig. 2F). Notably, the low levels of TIM3 reported for CD8^+^CD226^+^PD1^+^ TILs suggest that these cells do not display a terminally exhausted phenotype (Fig. 2F) [33].

Overall, these sets of experiments show that CD226 expression selectively decreased in CD8^+^ TILs isolated from liver metastases of CRC patients compared to matched peripheral blood lymphocytes, regardless of checkpoint inhibitor expression. The prognostic impact of CD226 expression was thus further investigated.

### The presence of CD8^+^CD226^high^ TILs in liver metastases is an independent prognostic determinant for CRC patient survival

To assess the prognostic impact of CD8^+^CD226^high^ TIL subsets in liver metastases, we first evaluated how the clinical and biological parameters of CRC contribute to CD8^+^CD226^+^ TIL infiltration. In general, clinical parameters, CD155 expression, and CD3 infiltration levels were not associated with CD226 expression on CD8^+^ TILs, though RAS mutations were associated with low CD226 expression by these cells (Table 1). The patients included in our cohort had liver mCRC and mainly displayed advanced CRC (84% of T3/T4) and lymph node involvement (64%). Most patients with mCRC underwent chemotherapy before resection (84%). Relapse after surgery mainly occurred in the lungs and the liver. Multivariate Cox regression analysis of RFS was performed (Table 2). Five parameters were identified in univariate Cox analysis as prognostic factors for RFS (*p* < 0.05): CD3 density measured in the tumor or at the invasion margin, CD8^+^ CD226^high^ T cells, CD4^+^ CD226^high^ T cells, and CD4^+^ TIGIT^high^ T cells (Table 2). In multivariate Cox analysis, three independent risk factors for RFS remained: CD3 density at the invasion margin (*p* = 0.003), CD226 expression on CD8^+^ T cells (*p* = 0.003), and TIGIT expression on CD4^+^ T cells (*p* = 0.019) (Table 2). Additionally, the coexpression status of CD226, CD96, TIGIT, PVRIG, CD112, and CD155 and their association with clinical parameters were analyzed using transcriptomic data (Supplementary Fig. 3), though no correlation was observed.

**Table 1:** Population Description

	CD226 High	CD226 Low	*p*-value		CD226 High	CD226 Low	*p*-value
**Sex -** ***n*** **(%)**				**Chemotherapy before resection -** ***n*** **(%)**			
Female	7 (18)	8 (21)	0.9	yes	16 (37)	20 (47)	0.11
Male	13 (33)	11 (28)		no	6 (14)	1 (2)	
**Age at metastasis resection**				**Nb of chemotherapy perfusion before resection**			
Median [1er quartile - 3e quartile]	67.5 [62–69]	60 [54–69]	0.41	Median [1er quartile - 3e quartile]	8 [6.75–11.25]	10.5 [7–13]	0.11
Min-Max	50–75	33–85		Min - Max	2–17	5–27	
**Metastatic at diagnosis -** ***n*** **(%)**				**Chemotherapy after resection -** ***n*** **(%)**			
Synchronous	10 (26)	14 (35)	0.23	yes	16 (37)	16 (37)	1
Metachronous	10 (26)	5 (13)		no	6 (14)	5 (12)	
**Localisation -** ***n*** **(%)**				**Relapse after surgery -** ***n*** **(%)**			
Colon	16 (41)	12 (31)	0.42	yes	13 (30)	16 (37)	0.38
Rectum	4 (10)	7 (18)		no	9 (21)	5 (12)	
**Primary tumor size -** ***n*** **(%)**				**Relapse site -** ***n*** **(%)**			
T4	3 (8)	4 (10)	0.19	Hepatic	7	13	
T3	16 (41)	10 (26)		Lung	7	10	
T2	0	2 (5)		Carcinosis	1	1	
NA	1 (3)	3 (7)		Bones	0	1	
**Primary lymph node invasion -** ***n*** **(%)**				**Histologic regression score (TRG) -** ***n*** **(%)**			
N0	5 (13)	4 (10)	0.99	1	1	2	0.86
N1	10 (26)	8 (21)		2	2	1	
N2	4 (10)	3 (8)		3	3	2	
NA	1 (2)	4 (10)		4	7	10	
**Primary histological differenciation -** ***n*** **(%)**				5	3	3	
Well differenciate	6 (15)	9 (23)	0.5	*Missing values*	6	3	
Moderate differenciation	12 (31)	9 (23)		**CD3 density in metastasis invasion margin**			
Missing values	3 (8)	0		Median [1er quartile - 3e quartile]	1020.5 [722.2–1486.1]	836.9 [529.2–1223.1]	0.36
**Microsatellite Instability -** ***n*** **(%)**				**CD3 density in metastasis tumor center**			
Stable	15 (38)	14 (36)	1	Median [1er quartile - 3e quartile]	307.15 [197.15–635.31]	203.8[86.81– 544.63]	0.13
Instable	1 (3)	0		**CD155 expression -** ***n*** **(%)**			
*Missing values*	4 (10)	5 (13)		0	9	8	0.56
**RAS mutation -** ***n*** **(%)**				1+	6	9	
Yes	7 (19)	15 (38)	0.005	2+	6	4	
No	12 (31)	2 (5)		3+	1	0	
*Missing values*	1 (2)	2 (5)		**Tumor infiltrative lymphocytes CD8 + **			
**RAF mutation -** ***n*** **(%)**				Median [1er quartile - 3e quartile]	36 [20–46]	45 [18–57]	0.43
Yes	0	0	0.72	**Tumor infiltrative lymphocytes CD4 + **			
No	17 (44)	15 (38)		Median [1er quartile - 3e quartile]	31 [17–57]	29 [21–49]	0.66
*Missing values*	3 (8)	4 (10)

**Table 2:** Multivariate Analysis

				Univariate analysis			Multivariate	analysis
	Number of patients	Number of events	HR	(95% CI)	*P*-value	HR	(95% CI)	*P*-value
**Tumor location**					0.240			
Colon	28	21	1					
Rectum	11	7	0.59	0.25–1.42				
**Histological grade**					0.129			
Well differentiated	15	14	1					
Moderately differentiated	21	11	0.54	0.24–1.20				
Missing values	3	3						
**Metastatic stage**					0.615			
Metachronous	15	8	1					
Synchronous	24	20	1.24	0.54–2.83				
**TRG**					0.542			
TRG 1-2-3	10	10	1.29	0.57–2.93				
TRG 4-5	20	14	1					
Missing values	9	4						
**Kras mutation**					0.180			
Wild type	14	10	1					
Mutated	22	17	1.74	0.78–3.91				
Missing values	3	1						
**Neoadjuvant chemotherapy**					0.401			
No	7	5	1					
Yes	32	23	1.53	0.57–4.09				
**Adjuvant chemotherapy**					0.704			
No	11	7	1					
Yes	28	21	1.18	0.50–2.81				
**CD3 intra tumoral**					**0.027**			0.670
Low	19	13	2.74	1.12–6.68		0.77	0.23–2.55	
High	20	15	1			1		
**CD3 invasion margin**					**<0.0001**			**0.003**
Low	19	15	8.92	2.74–29.05		9.62	2.11–44.00	
High	20	13	1			1		
**CD155 IHC**					0.592			
0	15	10	1					
+	14	10	1.51	0.61–3.73				
++/+++	10	8	1.53	0.58–4.06				
**CD8**					0.792			
Low	19	12	0.90	0.42–1.96				
High	20	16	1					
**CD8 CD226**					**0.003**			**0.003**
Low	19	16	3.50	1.55–7.90		3.90	1.60–9.53	
High	20	12	1			1		
**CD8 TIGIT**					0.640			
Low	19	11	0.83	0.38–1.83				
High	20	17	1					
**CD8 TIM3**					0.358			
Low	19	16	1.42	0.67–3.01				
High	20	12	1					
**CD8 PD1**					0.203			
Low	19	16	1.64	0.77–3.51				
High	20	12	1					
**CD4**					0.239			
Low	19	10	0.62	0.28–1.38				
High	20	18	1					
**CD4 CD226**					**0.030**			0.129
Low	19	16	2.37	1.09–5.15		1.90	0.83–4.33	
High	20	12	1			1		
**CD4 TIGHT**					**0.046**			**0.019**
Low	18	15	217	1.02–4.63		2.82	1.18–6.70	
High	21	13	1			1		
**CD4 TIM3**					0.942			
Low	19	14	1.03	0.48–2.19				
High	19	13	1					
Missing values	1	1						
**CD4 PD1**					0.175			
Low	19	16	1.73	0.78–3.82				
High	19	11	1					
Missing values	1	1						
**CD4 Th1**					0.973			
Low	19	13	1.01	0.48–2.15				
High	20	15	1					
**CD4 Th17**					0.847			
Low	18	14	1.08	0.50–2.34				
High	19	13	1					
Missing values	2	2						
**Treg**					0.703			
Low	5	4	1					
High	6	5	1.29	0.36–4.84				
Missing values	28	19						

**Fig. 3:**
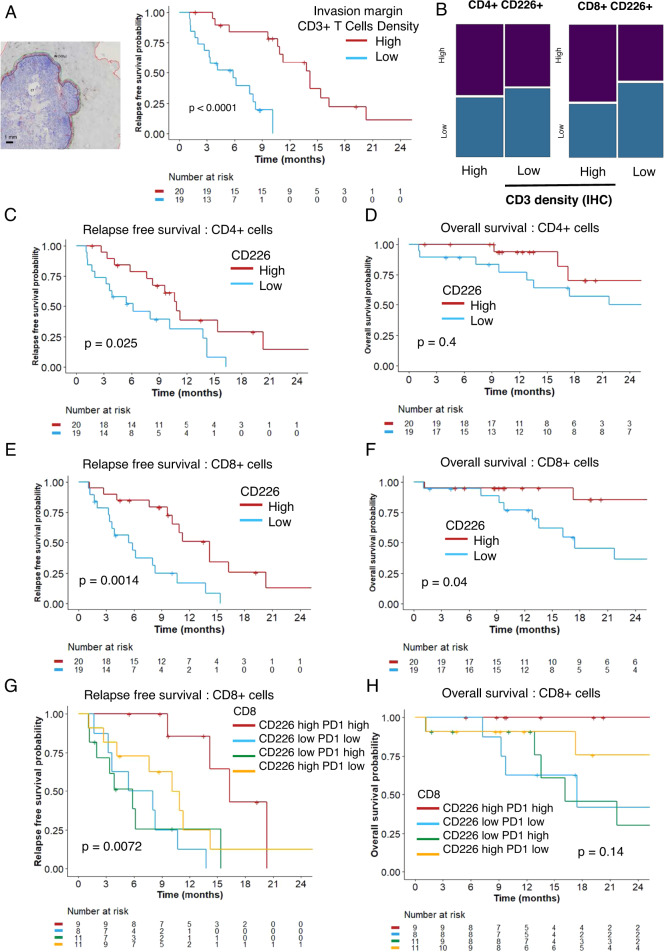
High CD226 expression on CD8^+^ T cells is associated with better survival. **A** Evaluation of CD3 with immunostaining and counting for the density in the tumor center and invasion margin with QuPath. The scale bar corresponds to 1 mm. RFS of 39 patients according to CD3 density in the tumor center, as discretized as high or low according to the median. **B** Comparison of CD226 levels, high or low, on CD4^+^ and CD8^+^, and CD3 density in the tumor center. CD3 density was discretized into two groups, high and low, according to the median density value of the full dataset. No significant difference was observed between the groups. **C** RFS according to median CD226 expression on CD4^+^ T cells discretized as high or low according to the median. **D** OS according to median CD226 expression on CD4^+^ T cells discretized as high or low according to the median. **E** RFS according to median CD226 expression on CD8^+^ T cells discretized as high or low according to the median. **F** OS according to median CD226 expression on CD8^+^ T cells discretized as high or low according to the median. **G** RFS according to CD8^+^ T cells positive or not for CD226 and PD1. Groups were discretized based on median expression. **H** OS according to CD8^+^ T cells positive or not for CD226 and PD1. Groups were discretized based on median expression. On survival graphs, p stands for the log-rank test. OS: overall survival; RFS: relapse-free survival

High CD3^+^ T cell density evaluated by immunohistochemistry in the central tumor zone (median RFS of 13,8 months [95% CI = 0.8 to NA months] versus 6.2 months [95% CI = 3.4 to NA months], *p* = 0.027) and in the invasion margin (median RFS of 14.2 months [95% CI = 10.9 to NA months] versus 5.9 months [95% CI = 3.3 to NA months], *p* < 0.0001) was associated with better RFS (Fig. 3A). However, high CD226 expression levels on CD4^+^ and CD8^+^ T cells did not correlate with CD3^+^ density in or around the tumor (Fig. 3B). Patients with high CD226 expression on CD4^+^ TILs had better prognosis in terms of RFS (median RFS of 10.9 months [95% CI = 8.3, NA months] versus 6.2 months [95% CI = 3.7 to 14.2 months], *p* = 0.025) but OS (median OS, 40.8 months [95% CI = 17.3, NA months], *p* = 0.4) (Fig. 3C, D). Regardless, patients with high CD226 expression on CD8^+^ TILs based on median expression had better RFS (median RFS of 14.2 months [95% CI = 10.1 to NA months] versus 5.4 months [95% CI = 3.4 to NA months], *p* = 0.0014) as well as OS (median OS of 40.8 months [95% CI = 40.8 to NA months] versus 17.4 months [95% CI = 13.6 to NA months], *p* = 0.04) (Fig. 3E, F). As suggested by multivariate analysis, additive prognostic value might involve monitoring CD226 expression on CD8^+^ T cells in patients with high CD3 infiltration. Indeed, in patients with high CD3^+^ lymphocyte infiltration, the median RFS was 11.5 and 15 months according to CD8^+^CD226^low^ and CD8^+^CD226^high^ T cell subsets, respectively. As PD1 is expressed by antigen-experienced T cells [33], the association between CD8^+^PD1^high^ and CD8^+^CD226^high^ and clinical outcomes was investigated, and better RFS and OS were observed in patients with PD1^−^ and CD226-enriched TILs (Fig. 3G-H).

Altogether, the ex vivo characterization of CD226 expression on CD8^+^ TILs performed in this validation cohort confirmed the results of RNA-seq in the UHB exploratory cohort. Thus, CD226 downregulation on CD8^+^ T cells is a prognostic biomarker associated with risk of relapse in patients undergoing surgery for CRC liver metastases. The prognostic value of the presence of antigens on PD1^high^ TILs is hampered when CD226 is downregulated on these lymphocytes.

### CD226 prognostic value is not CD155 dependent

**Fig. 4:**
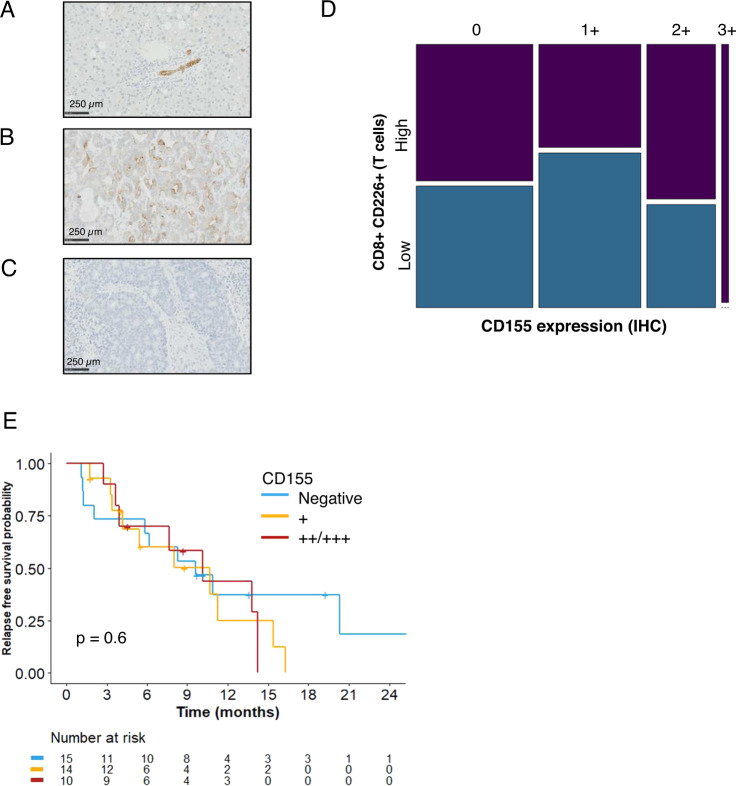
CD155 is not associated with CD226 expression or prognosis value. **A**–**C** CD155 immunohistochemistry (original magnifications x20) and scale bar corresponding to 250 µm. **A** Nontumor liver tissue with diffuse membranous positivity in the bile ducts. **B** CRC metastasis with diffuse membranous tumor cell positivity. **C** CRC metastasis without tumor cell staining. **D** Patient repartition according to level of CD226 on CD8^+^ T cells and CD155 staining of tumor cells. No significant differences were observed between the groups. **E** RFS after hepatic surgery according to CD155 expression on tumor cells.  + indicates weak expression, and + +/+++ indicates strong expression. On survival graphs, p based on the log-rank test. CRC Colorectal cancer; RFS Relapse-free survival

CD155-expressing CRC leads to better OS in primary colorectal (data not shown from TCGA) and breast cancers [34]. CD155 binds to CD226 and TIGIT to activate T cells and inhibit T cell function. The prognostic role of CD155 expression n CRC liver metastases has not yet been investigated and may be different from that suggested by primary CRC samples. Therefore, we investigated whether the presence of CD155 influences the prognostic value of CD226. For this purpose, CD155 expression was evaluated by immunohistochemistry and scored as 0 in the absence of expression. A score ranging from 1+ to 3+ according to staining intensity was used (Fig. 4A–C).

CD155 expression in liver metastases did not correlate with CD8^+^CD226^+^ T cell levels among TILs (Fig. 4D). Furthermore, expression of CD155 in liver metastases did not correlate with patient survival (Cox model, *p* = 0.6) (Fig. 4E).

In general, CD226 downregulation and the prognostic value of CD8^+^ TILs in liver CRC metastases do not depend on the presence of CD155.

### IL15 restores CD226 expression on CD8^+^ tumor-infiltrating lymphocytes and reverses CD8^+^ CD226^−^ T cell dysfunction

**Fig. 5:**
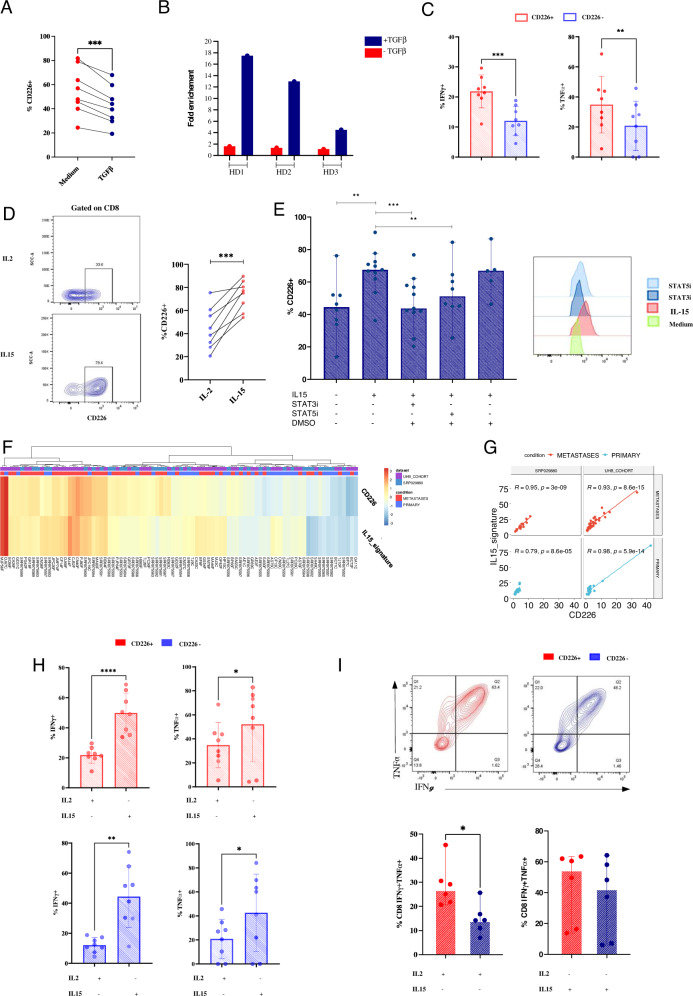
IL15 helps to restore CD226 expression on CD8^+^ TILs in liver metastasis. **A** Pooled data showing the effect of TGF-β1 on CD226 expression. **B** ChIP analysis of Smad2/3 binding to the CD226 promoter in CD8^+^ T cells stimulated or not with TGF-β1 (*n* = 5). **C** Intracellular production of IFNy and TNFα by CD226^+^ and CD226^–^ CD8^+^ TILs activated by anti-CD3-CD28. **D** CD226 expression on CD8^+^ TILs after 5 days of incubation with IL2 (control) or IL15. **E** Effect of STAT3 and STAT5 inhibitors (STAT3i and STAT5i, respectively) on CD226 expression by CD8^+^ T cells in the presence of IL15 **F**, **G** CD226 expression correlates with the IL15 signature in CRC liver metastasis and primary CRC based on bulk RNA-seq data. **H**, **I** Intracellular production of IFNy and TNFα by CD226^+^ and CD226^–^CD8^+^ TILs activated by anti-CD3-CD28 after 5 days of incubation with IL2 or IL15. Statistical differences between two groups were determined using the Mann‒Whitney U test or paired Student’s t-tests (**A, C, D, E, H, I**). *p < 0.05, **p < 0.01, ***p < 0.001. CRC Colorectal cancer

Because CD226 expression was dampened in liver metastases, in contrast to that in PBMCs of CRC patients, we hypothesized that tumor-derived immunosuppressive cytokines contribute to CD226 downregulation. To explore this, CD8^+^ T cells derived from PBMCs of healthy donors were isolated and exposed to TGFβ1. In this set of experiments, TGF-β1 significantly decreased CD226 expression by CD8^+^ T cells (Fig. 5A). To investigate the mechanism by which TGF-β decreases CD226 expression on CD8^+^ T cells, we performed ChIP (chromatin immunoprecipitation) analysis of Smad2/3 binding sites in CD226 promoter regions. The results showed an increased fold change in Smad2/3 binding to the CD226 promoter after TGF-β1 stimulation (Fig. 5B).

To address whether loss of CD226 expression on CD8^+^ TILs impedes their function, we performed TCR-induced activation using anti-CD3-CD28 microbeads and assessed intracellular production of IFNy and TNFα. Functional analysis revealed that CD8^+^ CD226^+^ TILs produced higher levels of IFNy than CD8^+^ CD226^−^ TILs (mean expression: 21% vs. 12%, *p* = 0.0002) (Fig. 5C). Moreover, CD8^+^ CD226^+^ T cells showed higher TNFα production capacity than their CD226^−^ counterparts (mean expression 34.8% vs. 20.8%, respectively, *p* = 0.003).

As IL15 has recently been shown to induce natural killer cell-associated activating receptors, we investigated the ability of this cytokine to restore CD226 expression on CD8^+^ TILs. For this purpose, TILs isolated from CRC liver metastases were stimulated for 6 days with IL15 or IL2 before characterization. Interestingly, IL15 significantly increased CD226 expression on CD8^+^ T cells compared to IL2 (mean expression 73% vs. 44.5%, *p* = 0.0008) (Fig. 5D). To confirm this finding, CD8^+^ CD226^−^ T cells were sorted and cultured with IL15. Consistent with our prior observations, IL15 indeed induced CD226 expression on CD8^+^ T cells (Supplementary Fig. 4A). We next investigated the molecular mechanisms by which IL15 increases CD226 expression on CD8^+^ T cells. Remarkably, CD226-induced expression was significantly abrogated in the presence of STAT3 and STAT5 inhibitors (*p* = 0.001 and *p* = 0.007, respectively) (Fig. 5E). These data suggest that IL15 induces CD226 expression on CD8^+^ T cells in a STAT3- and STAT5-dependent manner. The role of IL15 revealed through ex vivo experiments was supported by gene expression analyses, in which a correlation between the IL15 signature and CD226 expression levels was observed in both primary and liver metastases of CRC (Fig. 5F, G).

IL15 has been demonstrated to enhance the antitumor response of cytotoxic T cells [35], and we thus assessed the role of IL15 in regulating CD226^+^ and CD226^−^ T cell responses. Functional analysis showed enhanced capacity for IFNy (mean expression 21% vs. 49%, respectively, *p* < 0.0001) and TNFα (mean expression 34% vs. 52%, respectively, *p* = 0.04) production following IL15 stimulation by CD226^+^ T cells compared to IL2 (Fig. 5H). CD8^+^ CD226^+^ T cells showed increased production of TNFα compared to CD226^−^ T cells, but IL15 restored CD226^−^ T cell capacities of IFNy and TNFα production (Fig. 5H, Supplementary Fig. 4B). Although IL15 restored CD226^−^ T cell dysfunction, interestingly, CD226^+^ T cells exhibited more polyfunctional traits than CD226^−^ T cells (Fig. 5I).

## Discussion

In this study, we demonstrate the prognostic impact of CD226, as evaluated by flow cytometry using freshly extracted, noncultured CD8^+^ TILs from liver metastasis of CRC, which guaranteed the accuracy of the expression data for the studied biomarkers. Patients with CD8^+^CD226^high^ T cells had better survival and lower relapse rates after liver metastasis resection than those with CD8^+^CD226^low^ TILs. This is consistent with the association between CD226 expression and potent immune infiltration in the tumor microenvironment. Moreover, CD226 seems to correlate better with strong immune activation in liver metastasis than in primary CRC, despite the known immunosuppressive tumor microenvironment (TME) in the liver and the inflammatory context in primary CRC.

CD226 was not associated with any clinical feature except for RAS mutation. CD226^high^ T cells were enriched in patients with nonmutated RAS, which is consistent with preclinical models in which KRAS mutations were associated with immunosuppressive chemokines, such as IL10 and TGFβ, leading to CD8^+^ T cell depletion and a reduction in PD1 expression in these cells [36]. Overall, RAS-mutated tumors are associated with MHC class I loss and decreased T cell activation [37].

Survival analysis revealed that PD1^+^CD226^+^CD8^+^ TILs were associated with better RFS and OS for CRC-LM patients. It is now well established that PD1 expression on TILs is a double-edged sword when predicting prognosis in cancer patients, as its expression has been associated with both unfavorable [38–40] and favorable [41, 42] clinical outcomes. PD1 has been widely considered a T cell exhaustion biomarker associated with poor prognosis, yet the presence of PD1 on lymphocytes also indicates previous antigen exposure and the presence of antigen-experienced T cells. Moreover, PD1 expression correlates with oligoclonal expansion of tumor-reactive T cells [43, 44]. Interestingly, the correlation between PD1^+^CD226^+^CD8^+^ TILs and better RFS supports the latter hypothesis. Few studies have investigated the prognostic value of CD226 expression on CD8^+^ TILs [18–20]. Most studies have focused on the predictive value of CD226 expression in checkpoint inhibitor blockade. However, Mellman et al. showed that PD1 inhibits CD226 phosphorylation via its ITIM-containing intracellular domain [19]. CD226 expression is also associated with clinical benefits in patients with non-small cell lung carcinoma treated with anti-PD-L1 [19]. In pancreatic cancer, high CD226 expression on CD8^+^ T cells is associated with response to PD1 and TIGIT blockade [15].

Recently, a study conducted by Martinet et al. [18] demonstrated that loss of CD226 expression is associated with a dysfunctional state of CD8^+^ TILs due to impaired TCR signaling in the absence of CD226. Consistent with these results, we showed that following TCR stimulation, CD226^−^CD8^+^ TILs in metastatic CRC were hyporesponsive compared to CD226^+^CD8^+^ TILs.

Despite the established role of CD226 in antitumor immunity, the mechanisms underlying its regulation in the TME remain elusive. Interestingly, our results showed decreased expression of CD226 on TILs compared to matching PBMCs. This observation led us to hypothesize that once in the TME, CD226 expression on tumor cells is downregulated. Although CD155 has been demonstrated to play a role in CD226 loss of expression via proteasomal degradation of CD226 [20], our results indicated no correlation between CD155 and CD226 expression in CRC with liver metastasis, suggesting the existence of other mechanisms that drive loss of CD226 expression in the TME. The impact of BCL9 suppression on promoting CD155 expression by tumor cells was recently demonstrated, providing new insight into the role of BCL9 in regulating CD226 and CD96 immune receptors [45]. Accordingly, we investigated the role of TGF-β in CD226 regulation. Interestingly, TGF-β significantly decreased CD226 expression on CD8^+^ T cells, and ChIP analysis supported the hypothesis that TGF-β might downregulate CD226 expression in a Smad2/3-dependent fashion. Next, we investigated how to restore CD226 expression in TILs and whether it affects TIL functions. As IL15 induces expression of CD226 on NK cells [14], we sought to investigate whether it helps to restore CD226 expression on CD8^+^ TILs. In this study, use of IL15 significantly increased CD226 expression on CD8^+^ TILs, and IL15 significantly increased CD226 expression on CD8^+^ TILs in a STAT3/5-dependent manner. Moreover, IL15 enhanced CD226^+^ CD8^+^ TIL functions and helped to restore CD226^−^ TIL functionality. Our results led us to hypothesize that use of IL15 in combination with immunotherapies might help to restore CD226-deficient TIL antitumor capacities [19].

In summary, CD226 selectively contributes to the immune surveillance of liver metastasis of CRC. Patients who undergo surgery for CRC liver metastasis have longer survival times; however, there is a need to identify patients who require treatment intensification to achieve better survival. Our results show that CD226 can be used as a biomarker for stratifying patients with resected liver metastasis for complementary treatment and surveillance according to their prognosis. Our findings also highlight the importance of IL15 use for CD226 expression and T cell functionality restoration and that this cytokine may be included in immunotherapeutic strategies to increase tumor control.

## Supplementary information

Supplementary Figure 1

Supplementary Figure 2

Supplementary Figure 3

Supplementary Figure 4

Supplementary figure legend

supplementary methods

## Data Availability

The data analyzed in this study were obtained from Gene Expression Omnibus (GEO) at GSE50760, GSE14333, GSE17536, GSE33113, GSE37892, GSE39582, SRP060016, and GSE178318. The data generated in this study are publicly available in the Gene Expression Omnibus (GEO) database under GSE207194.
