# Supplementary figures and images for: CD8+ CD226high T cells in liver metastases dictate the prognosis of colorectal cancer patients treated with chemotherapy and radical surgery
Source: Cell Mol Immunol. 2023 Jan 30;20(4):365–78. doi: 10.1038/s41423-023-00978-2 (PMC10066387; doi:10.1038/s41423-023-00978-2)

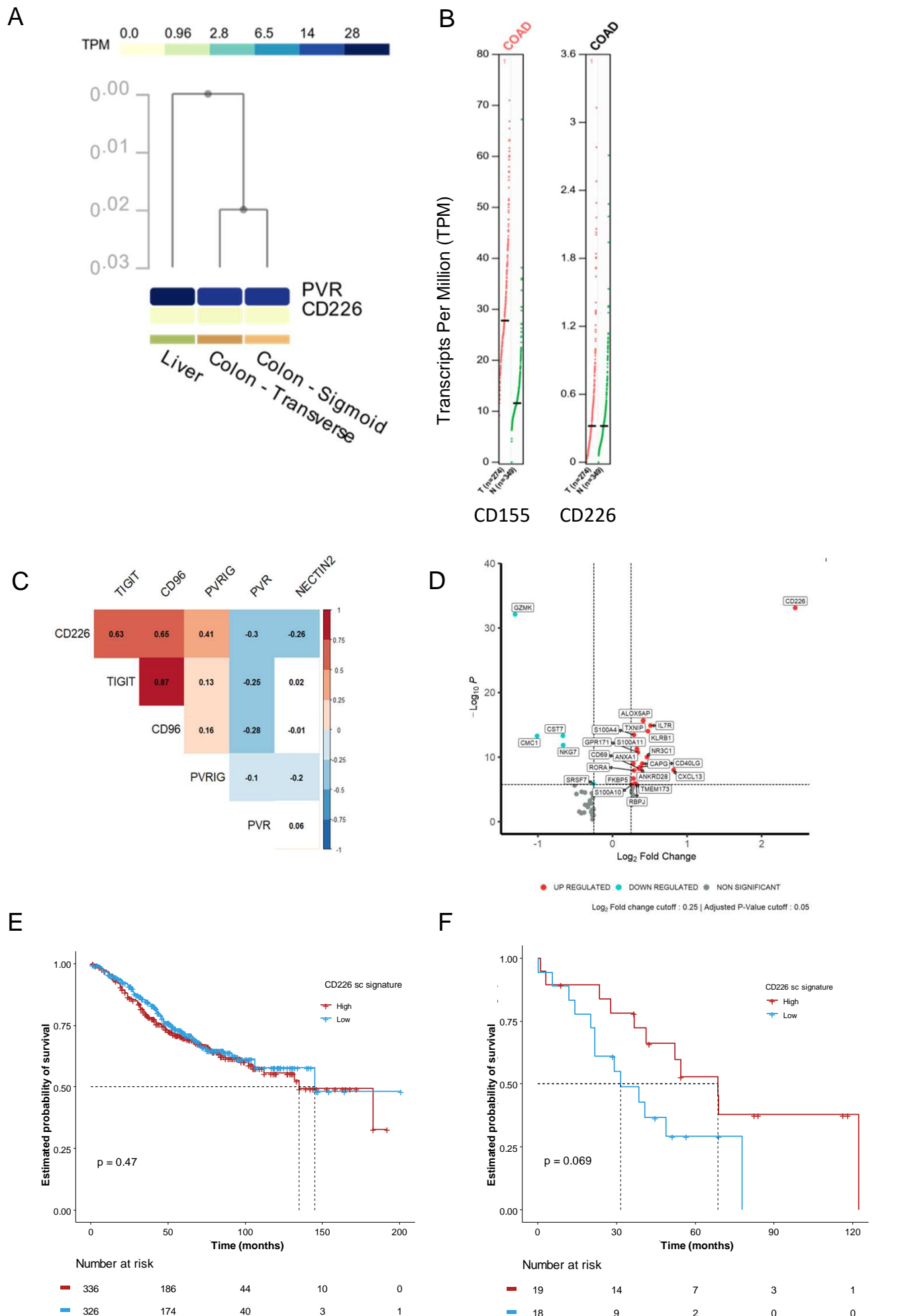

Supplementary Figure 1

Supplement: Supplementary file 1 — Supplementary Figure 1 [file 41423_2023_978_MOESM1_ESM.pdf]

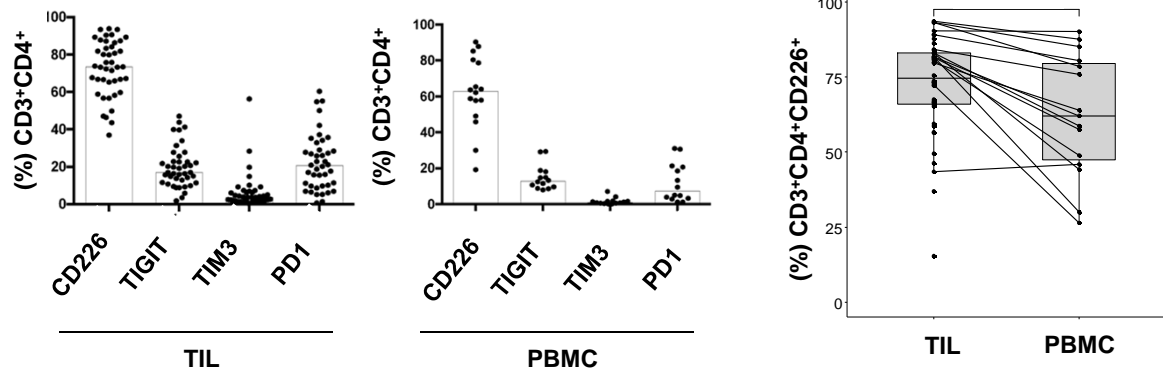

Supplement: Supplementary file 2 — Supplementary Figure 2 [file 41423_2023_978_MOESM2_ESM.pdf]

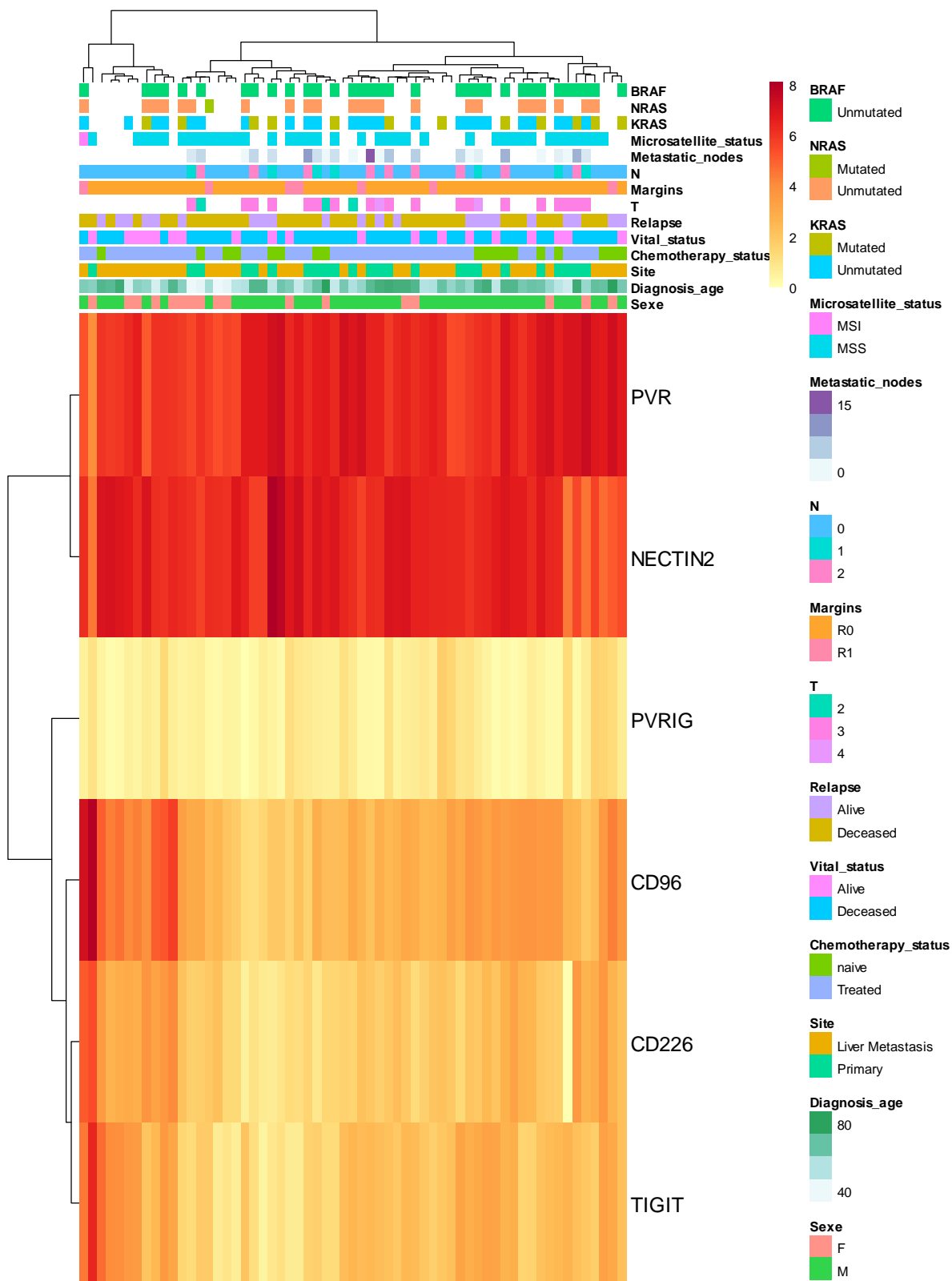

Supplementary Figure 3

Supplement: Supplementary file 3 — Supplementary Figure 3 [file 41423_2023_978_MOESM3_ESM.pdf]

A

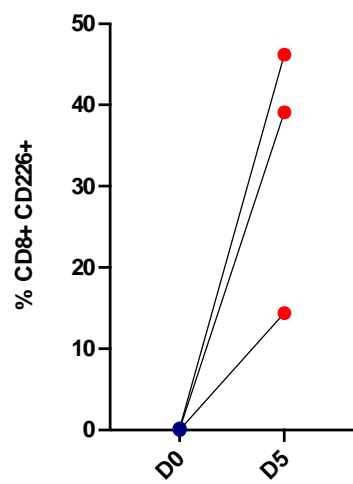

B

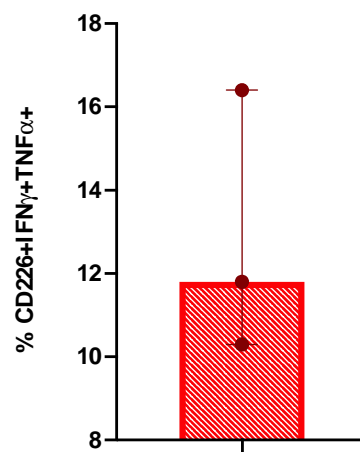

Supplement: Supplementary file 4 — Supplementary Figure 4 [file 41423_2023_978_MOESM4_ESM.pdf]
