# Supplementary material for: CD8+ CD226high T cells in liver metastases dictate the prognosis of colorectal cancer patients treated with chemotherapy and radical surgery
Source: Cell Mol Immunol. 2023 Jan 30;20(4):365–78. doi: 10.1038/s41423-023-00978-2 (PMC10066387; doi:10.1038/s41423-023-00978-2)
Supplement: Supplementary file 5 — Supplementary figure legend [file 41423_2023_978_MOESM5_ESM.pdf]

CD8^{+} CD226^{high} T cells in liver metastases dictate the prognosis of colorectal cancer patients treated with chemotherapy and radical surgery

Supplementary Figure 1. Correlation matrix for ligands and receptors from the CD226 axis and survival analysis in primary CRC

A. CD226 and his ligand CD155 (PVR) mRNA level expression in normal tissu from GTEx. **B**. CD226 and his ligand CD155 mRNA expression level comparison between normal adjacent tissu and tumoral primary CRC. **C**. Correlation matrix from colorectal gene expression (FPKM) from TCGA. Significant correlation marked in color gradient according to their value. Non-significant association are in white. $p<0.05$ was considered significant. **D**. Volcano Plot of the gene differential expression between CD226^{+} and CD226^{-} T cells from CRC, including primaries and liver metastases. **E**. Estimated probability of survival according to the CD226 signature created from the previous differential analysis in a *« home curated »* database of primary CRC. Score was calculated from the geometric mean of the mRNA expression level from the gene list. The survival analysis was divided in two based on the median value of all CD226 signature scores. **F**. Same analysis starring UHB cohort of resected CRC liver metastasis.

On survival graphs, p stand for the log-rank testing.

CRC: colorectal cancer

Supplementary Figure 2. Description of CD226 expression in TILs and matched peripheral T lymphocytes

Checkpoints expression on CD4^{+} T cells in TILs and PBMC. Percentage of CD4^{+} T cells positives for the receptors are represented on the left. On the right, paired plot representing percentage of CD3^{+}CD4^{+}CD226^{+} cells between TILs and PBMC.

Supplementary Figure 3. Correlation between clinical parameters and CD226 axis ligand/receptors (UHB cohort)

Heatmap representing RNA expression with TMM normalization and log2(x+1) transformation for ligands and receptors associated with CD226 axis. Available clinical annotations for each

patient represented as a column are reported on top of the heatmap. Missing annotations are reported as white.

Supplementary Figure 4: IL15 induces CD226 expression on sorted $CD8^+CD226^-$ T cells

A. 5 days culture of sorted $CD226^-$ $CD8^+$ T cells with IL15 induces CD226 expression. **B.** Intracellular production of IFNγ and TNFα by IL15-induced CD226+ $CD8^+$ T cells activated by a-CD3-CD28.
