# Supplementary material for: CD8+ CD226high T cells in liver metastases dictate the prognosis of colorectal cancer patients treated with chemotherapy and radical surgery
Source: Cell Mol Immunol. 2023 Jan 30;20(4):365–78. doi: 10.1038/s41423-023-00978-2 (PMC10066387; doi:10.1038/s41423-023-00978-2)
Supplement: Supplementary file 6 — supplementary methods [file 41423_2023_978_MOESM6_ESM.docx]

CD8^+^ CD226^high^ T cells in liver metastases dictate the prognosis of colorectal cancer patients treated with chemotherapy and radical surgery

**Supplementary Methods:**

**Human tumor processing for TILs extraction**

A single tumor sample was cut into small fragments of 2–3 mm in length. The fragments were then subjected to mechanical/enzymatic dissociation (Miltenyi Biotec). Briefly, the fragments were placed in a C-tube (Miltenyi Biotech) with an enzyme digestion mix consisting of RPMI 1640 (Gibco), 1 mg/mL collagenase, and 30 U/mL DNAse (both from Sigma-Aldrich). Fragments were incubated for one hour at 37°C interspersed by three 36-second mechanical disaggregation steps (programs h_tumor_01.01, 02.01, and 03.01) performed in the GentleMACS dissociator (Miltenyi Biotec) just before, 30 minutes and one hour after the beginning of enzymatic digestion. Following tumor digestion, the cell slurry was passed through 70 m and 30 m sterile strainers (Miltenyi Biotec) to remove undigested tissue chunks. The resulting cell suspension was washed twice with PBS and subjected to immunofluorescence staining for flow cytometry analysis. No attempt was made to separate the tumor-infiltrating lymphocytes from the tumor cells to avoid cell loss, which could distort the proportions of various mononuclear cells present within the infiltrate.

**Immunohistochemistry:**

CD3 slides were deparaffinized using EZ Prep (Ventana, Roche). Antigen retrieval was performed by incubating the slides for 60 minutes in CC1 buffer (Ventana, Roche). Tissue sections were then incubated with a polyclonal rabbit anti-human CD3 antibody (A045201, Agilent) [1:200] for 32 min. The reaction was performed using the UltraView Universal DAB Detection Kit (Roche). Finally, the sections were counterstained with hematoxylin, dehydrated, and mounted. CD3 slides were digitized with a Nanozoomer HT2.0 (Hammamatsu) at ×20 magnification to generate a whole slide imaging (WSI) file in ndpi format. All WSI files were imported into the QuPath software (26). A pathologist manually contoured the tumor area to define the centrotumoral zone (CT) and invasive margin (IM). IM was defined as a band of 500µm around the tumor area with an internal marge of 500µm. A script automatically generated these two areas from the contoured zone, and then, using QuPath detection scripting, positive cells were differentiated from negative ones to evaluate densities (cells/mm²) of CD3 in CT and IM.

For CD155 immunohistochemistry, formalin-fixed, paraffin-embedded tissues were cut into 5-μm sections, deparaffinized, and rehydrated in a graded series of ethanol. Antigen retrieval was performed by heating the tissue sections using the Target Retrieval Solution (pH 6.0, DAKO). To block endogenous peroxidase, the sections were immersed in a 3% solution of hydrogen peroxide (ready to use) for 15 min at room temperature and then washed in phosphate-buffered saline for 10 min. pAb against CD155 (anti-poliovirus/PVR, Abcam) was added and the sections were incubated for 1 h at room temperature. The sections were then washed twice in phosphate-buffered saline containing 0.4% Tween for 5 min each, and EnVision+ Rabbit/HRP (DAKO) was used according to the manufacturer’s instructions. The reaction products were visualized using 3,3’-diaminobenzidine tetrahydrochloride, and the sections were counterstained with Mayer’s hematoxylin and coverslipped.

CD155 expression was performed at x200 magnification. CD155 expression was defined as membranous staining of tumor and bill duct cells. The histological sections were classified into two categories as follows: specimens with CD155 positive tumor cells were classified as CD155 positives and others as CD155 negatives.

**Bioinformatic analysis**

Gene expression in normal tissues was analyzed using the GTEx portal website. The GEPIA web interface was used to compare the expression in normal and cancer tissues.
